# Full green assay of parenteral dosage forms of polymyxins utilizing xanthene dye: application to content uniformity testing

**DOI:** 10.1186/s13065-024-01261-9

**Published:** 2024-08-27

**Authors:** Mahmoud A. Abdelmajed, Khalid M. Badr El-Din, Tamer Z. Attia, Mahmoud A. Omar

**Affiliations:** 1 Department of Pharmaceutical Analytical Chemistry, Faculty of Pharmacy, Deraya University, New Minia, Egypt; 2 Department of Analytical Chemistry, Faculty of Pharmacy, Minia University, Minia, Egypt; 3 Department of Pharmacognosy and Pharmaceutical Chemistry, College of Pharmacy, Taibah University, Medinah, Saudi Arabia

**Keywords:** Polymyxins, Colistin (polymyxin E), Polymyxin B, Erythrosine B, Parenteral dosage forms, Content uniformity testing

## Abstract

Due to the lack of other treatment options, a rebirth of polymyxins is urgently required. Colistin (also called polymyxin E) and polymyxin B are the only two examples of this antibiotic class that were effectively employed in such critical situations. In the present work, both of the two studied medications were quantified via a simple, green, and non-extracting spectrophotometric approach based on the formation of ion-pair complexes with Erythrosine B. Without using any organic solvents, the pink color of the created complexes was detected at wavelength = 558 nm. To achieve the highest intensity of absorbance, optimum conditions were established by the screening of many experimental factors such as pH, buffer volume, the volume of Erythrosine B, and the time consumed to undergo the reaction. For Colistin and Polymyxin B respectively, Beer-Lambert’s law was observed at the concentration ranges of 1–6, 1–9 µg mL^− 1^. The technique was approved and validated following ICH recommendations. Lastly, the suggested approach has been successfully implemented to quantify the cited medications colorimetrically, for the first time, in their parenteral dosage forms with excellent recoveries. Also, Content uniformity testing was implemented.

## Introduction

**Fig. 1:**
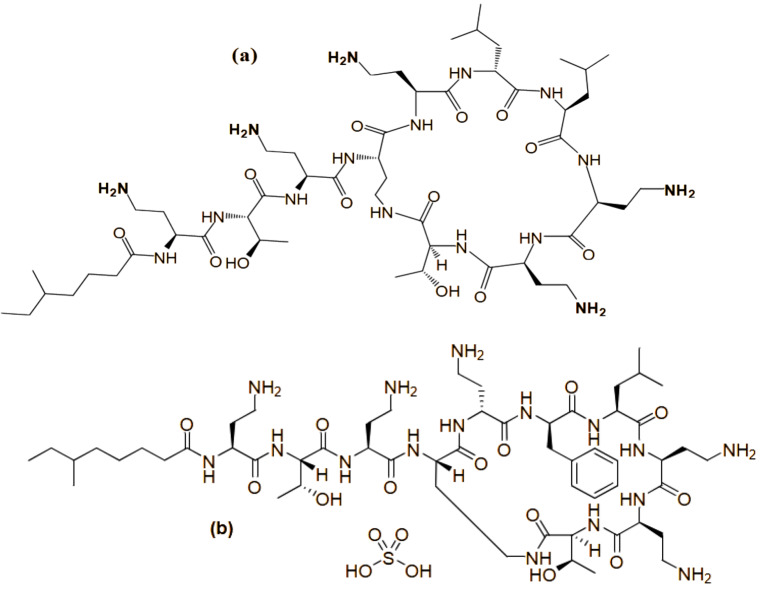
Chemical structure of Colistin **(a)** and Polymyxin B **(b)**

With the emergence of multidrug-resistant (MDR) Gram-negative bacteria and the lack of innovative antimicrobial medications, the revival of polymyxins (PMS) is urgently essential [1, 2]. Recently, there has been a significant rise in infections caused by MDR Gram-negative pathogens, named Pseudomonas aeruginosa, Acinetobacter baumannii, and Klebsiella pneumoniae. For these subtypes of bacteria, PMS are frequently the only active antibiotics and are remarkably successful in killing them. Colistin (CS) and Polymyxin (Poly B) have relatively similar chemical structures. The only difference between the two structures occurs in one amino acid at position 6 of the peptide ring, where CS has a d-leucine and Poly B has a d-phenylalanine, as observed in (Fig. 1). Moreover, the way that both drugs are delivered into the human body is also one of their main differences. While Poly B is given directly as its active form, CS is taken as an inactive less toxic prodrug called Colistimethate Sodium (CMS) [3].

Numerous analytical strategies based on spectroscopic (spectrophotometric [4–8] and fluorimetric [9–14] methods and also microbiological assays [15–19] were reported. On the other hand, chromatographic approaches include high-performance liquid chromatography (HPLC) [20–26], liquid chromatography coupled with mass detector (LC-M) [27–36], and capillary electrophoresis (CE) [37–40], also published. As well known, separative techniques such as HPLC and LC-M consume large amounts of energy, need well-experienced operators, and have complex and costly instrumentation. Microbiological assays need a long incubation time and lack selectivity and sensitivity. Belongs to the reported spectrophotometric approaches [5–8] were less sensitive than the present work. Also, the complex formation with FeCl_3_ [4] needed an organic solvent (methanol) but the present study has been completely performed in aqueous media. Related to the fluorimetry technique, three articles were laborious and time-consuming [9–11]. And the other needed a costly reagent and organic solvent (acetone) to proceed [12]. Consequently, the valuable aim of the current work was to establish a rapid, sensitive, cost-effective, and green alternative technique for analyzing these drugs. Moreover, it is the first colorimetric paper employed to quantify the parenteral dosage forms of the investigated drugs, along with the employment of Content uniformity testing and greenness evaluation.

Ion pair complex formation is one of the most applied spectroscopic approaches that depend on the colored product measurement in the visible region. Therefore, the final colored product is devoid of any interfering substances such as any solvent or degradation product. Erythrosine B (EB) is a xanthene dye that is widely used as a spectroscopic probe in the investigation of pharmaceutical and biological substances [41]. It is one of the most commonly employed reagents in ion-pair complex reactions in a simple methodology. Chemically, it is a disodium salt of 2, 4, 5, 7-tetraiodofluorescein having a negative charge that contributes to the formation of the colored complex together with the positive charge of the experimental substances.

## Experimental

### Devices

All colorimetric measurements were performed using a spectrophotometer made by Shimadzu UV-visible 1900i (Tokyo, Japan). Two quartz sample cells (1 cm) were utilized to measure the absorbance. A digital analytical balance (Mettler Toledo, Glattbrugg, Switzerland), a thermostatically controlled MLW type water bath (Memmert Gmbh, Schwabach, Germany), and an ADWA 11 pH meter (Romania) instruments also were involved in the described methodology.

### Pure powder and marketed formulations

CS and Poly B powders with purity grades of 99.2% and 98%, respectively were kindly gifted by the National Organization for Drug Control And Research (NODCAR) and utilized without further purification.

Paximid^®^ vial (a Cipla Company product) is labeled to contain 500,000 IU, which is equal to 5 mg of the authentic drug). Colomycin^®^ lyophilized powder for injection (Forest Laboratories, United Kingdom) is labeled to contain 2 M.I.U which is equivalent to 160 mg of CMS, the prodrug of CS.

### Chemicals and reagents

EB was obtained from (Loba Chemie, Mumbai, India) and prepared as 0.15% (w/v) in distilled water (DW).

### Drug stock solution

CS and Poly B were daily prepared as a working solution of 100 µg mL^− 1^ by weighing 10 mg of each authentic in 100 mL of DW.

### Standard analytical procedure

In addition to one mL of the screened medications’ working solutions, one mL of acetate buffer (pH = 4) was added to a 10-mL volumetric flask. Then 0.5 mL of 0.15% w/v EB was poured. The flasks’ components were kept at room temperature for ten minutes. Eventually, following the dilution with DW, the absorbance of each medication was determined at a wavelength of 558 nm. A blank solution was made in the same way without the addition of the cited drugs.

### Quantification of CS and poly B in their Market products

#### Preparation of vial form of the studied drugs

A precise amount from Colomycin^®^ vial equal to 10.0 mg of the studied medication was poured into a volumetric flask of 100 mL, then five mL of 0.2 M sulfuric acid was added and remained for ten minutes to convert all CMS into CS [42]. Furthermore, ten mL of 0.2 M NaOH was added to neutralize the solution. Belongs to Poly B, in a 100-mL standard flask, an exact equivalent to 10 mg of Poly B was moved and completed to mark with DW. Then, further dilution was employed to obtain working solutions and previously general analytical procedures were implemented for both drugs.

#### Preparation of Colistin Sulfate^®^ tablets

Twenty tablets were weighed, ground, and well-mixed. After that, an aliquot amount equal to 10.0 mg of CS was mixed with 30 mL of D.W. and sonicated for 15 min. Filtration and further dilution were employed to provide the final working solutions. Finally, the general analytical procedure was followed.

#### Procedure for Content Uniformity Testing

During the investigation, 10 tablets of Colistin sulfate^®^ were individually well-shriveled, from each crushed pill, a specified quantity equal to 10 mg of authentic was employed and then followed the same steps in Sect. 2.6.2. Testing for content homogeneity was carried out following USP requirements [43]. The acceptance value (AV) was determined after 10 tablets had each been evaluated independently.

## Results and discussion

**Fig. 2:**
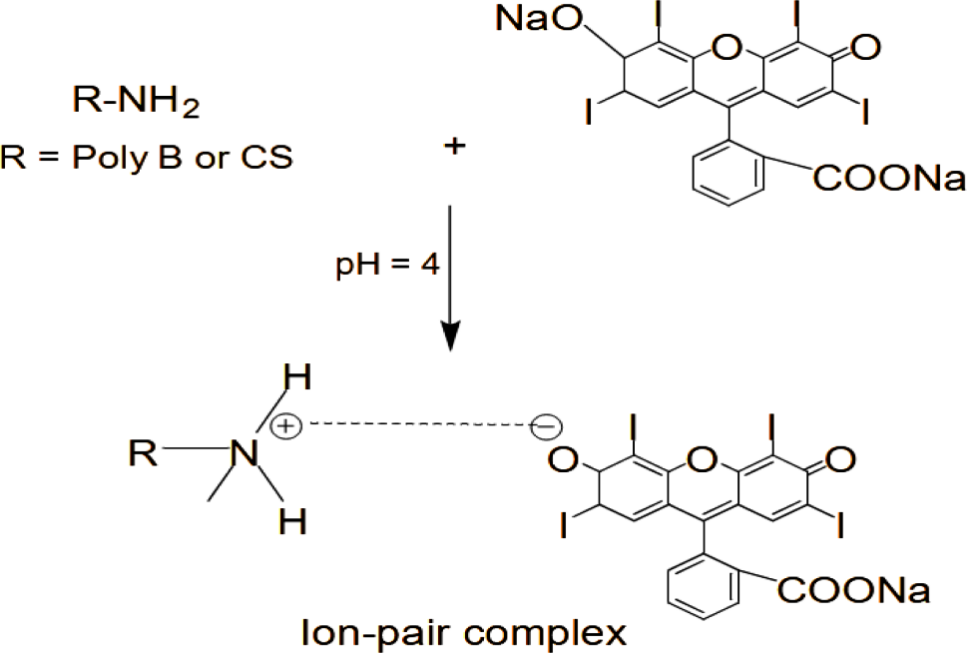
Schematic pathway between polymyxins and Erythrosine B

**Fig. 3:**
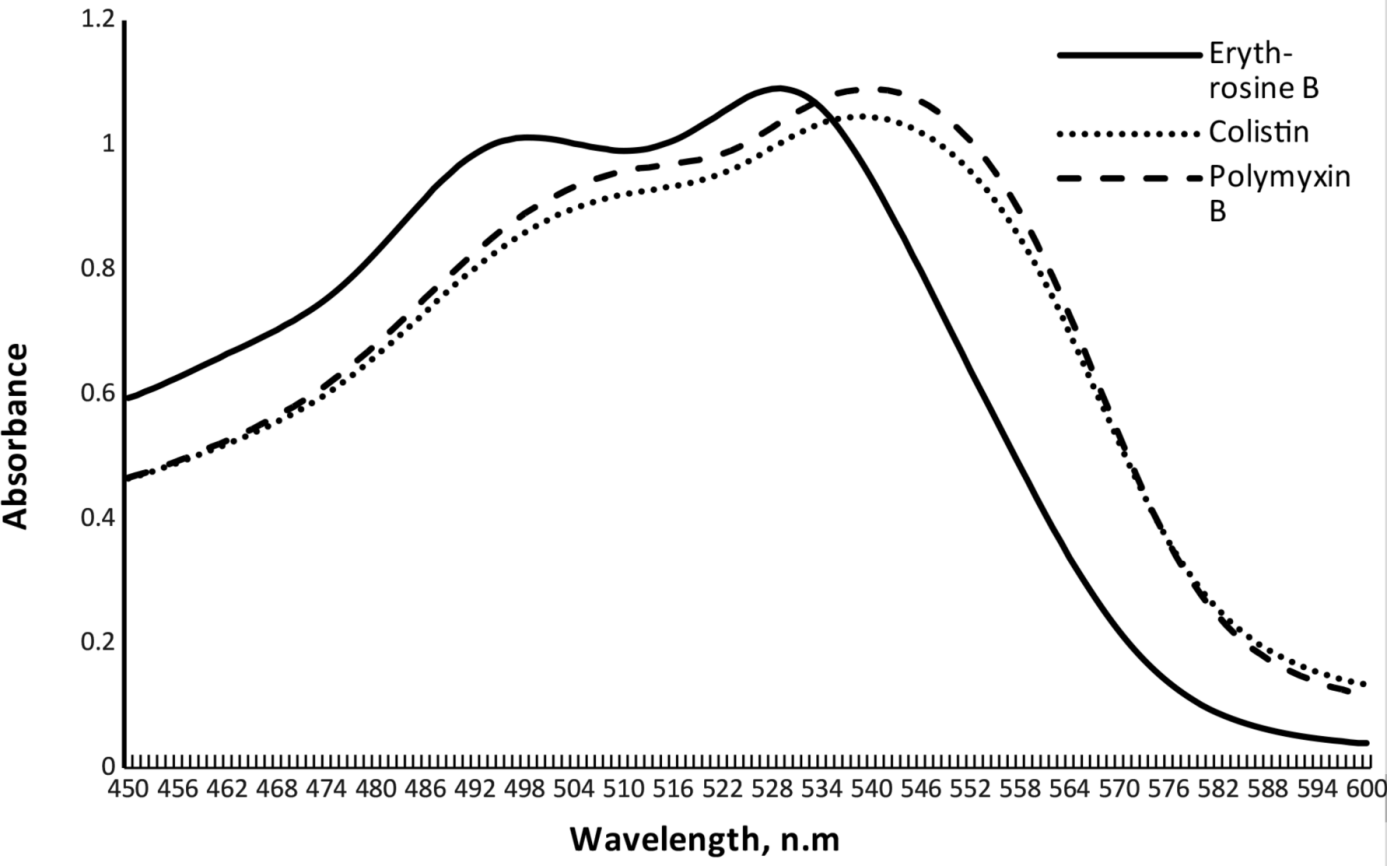
Absorption spectra of ion-pair complexes of Colistin (6 µg mL^− 1^) and polymyxin B (9 µg mL^− 1^) with Erythrosine B

The formed products were water-soluble and no organic solvents were involved throughout the described approach. Moreover, the resultant ion-pair complexes were measured precisely and directly without the necessity for organic solvent extraction. All these features conflict with the environmental friendliness, simplicity, and not time-consuming of the present work over the other reported methods. Several medications were spectrophotometrically and spectrofluorimetrically investigated through complex formation with EB [44–50]. In an acidic media, the reaction between CS and Poly B produced a stable, robust, and water-soluble pink complex with a maximum absorbance at λ = 558 nm. The schematic reaction pathway and approach’s spectrum were shown in (Fig. 2) and (Fig. 3) respectively.

### Optimization

To find the best reaction circumstances required to produce the highest absorbance readings, certain technique factors were varied while the others remained frozen.

#### Buffer solution

**Fig. 4:**
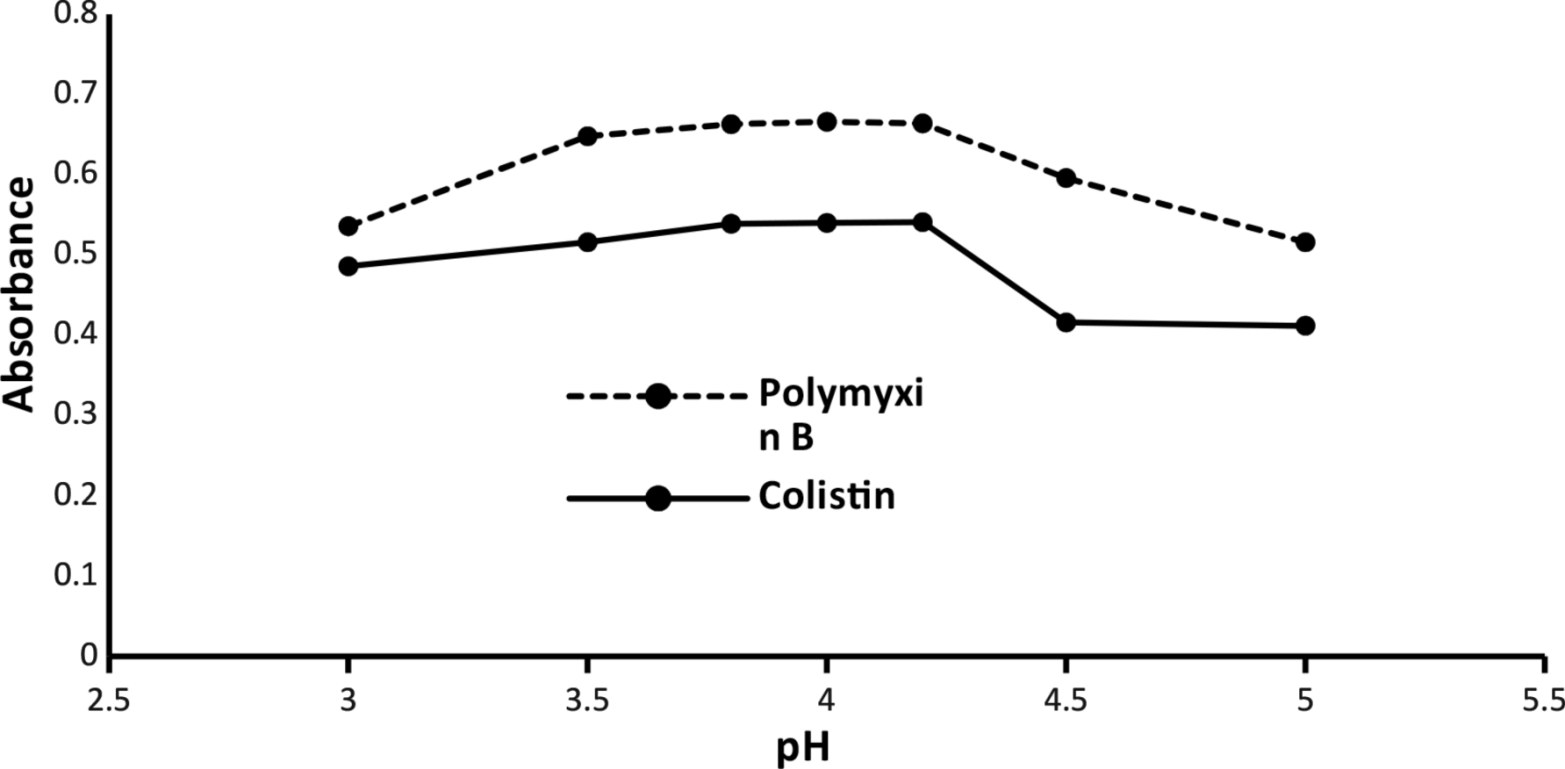
Effect of the pH on the absorbance of the ion-pair complexes of Colistin (3 µg mL^− 1^) and Polymyxin B (5 µg mL^− 1^)

**Fig. 5:**
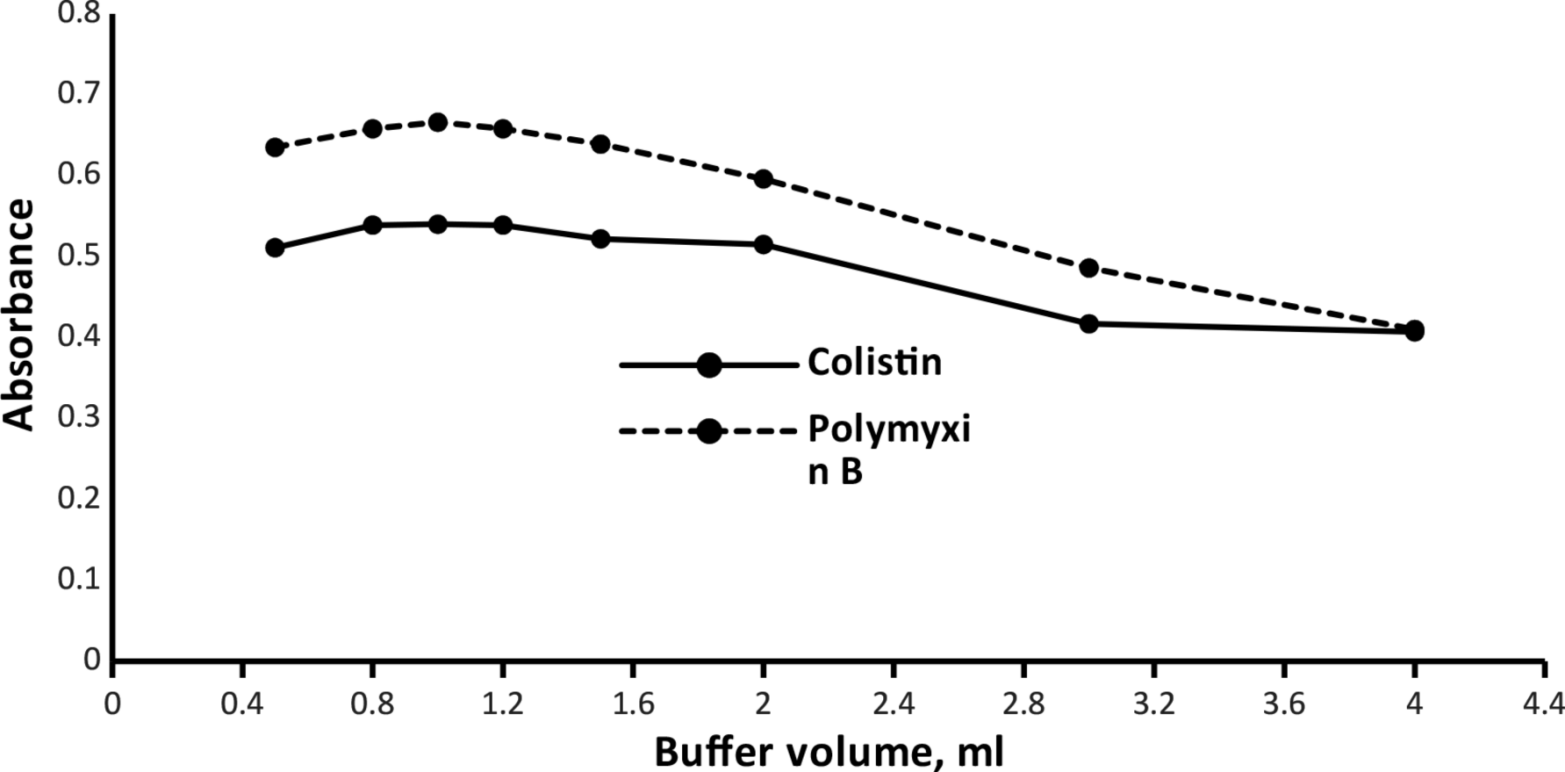
Effect of the buffer volume on the absorbance of the ion-pair complexes of Colistin (3 µg mL^− 1^) and Polymyxin B (5 µg mL^− 1^)

Because any alteration in pH has a significant effect on the yielded absorbance of the colored complex, a buffer of acetate was utilized to obtain a pH range (3–5). The best values were in the pH range (3.8–4.2) and any deviation had an extremely bad effect on the outcomes. During the screening of the optimal volume of the used buffer, the maximum ABS has been recorded between the quantities (0.5–1.5 mL). As a result, 1 mL acetate buffer (pH 4) was the best buffer condition, as shown in (Figs. 4 and 5).

#### Erythrosine B volume optimization

**Fig. 6:**
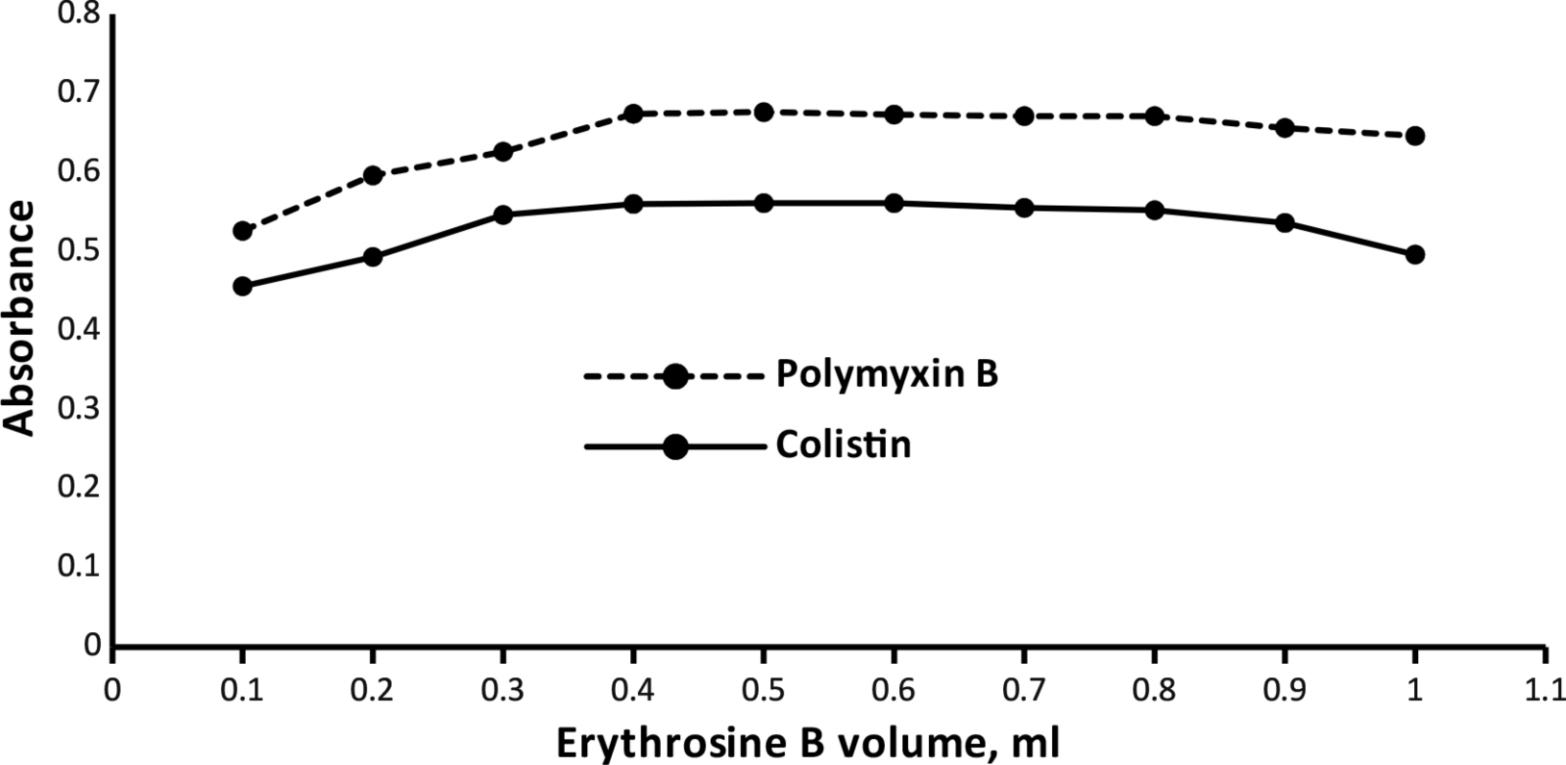
Effect of erythrosine B volume on the absorbance of the ion-pair complexes of Colistin (3 µg mL^− 1^) and Polymyxin B (5 µg mL^− 1^)

The cited drugs were subjected to varying volumes of the dye ranging from (0.1-1 mL). Turbidity in the experimental solutions was caused by higher dye volumes. Consequently, 0.5 mL of 0.15% w/v EB for both drugs yielded the most stable complexes with the best values of ABS. All details have been illustrated in (Fig. 6).

#### Reaction time and temperature optimization

**Fig. 7:**
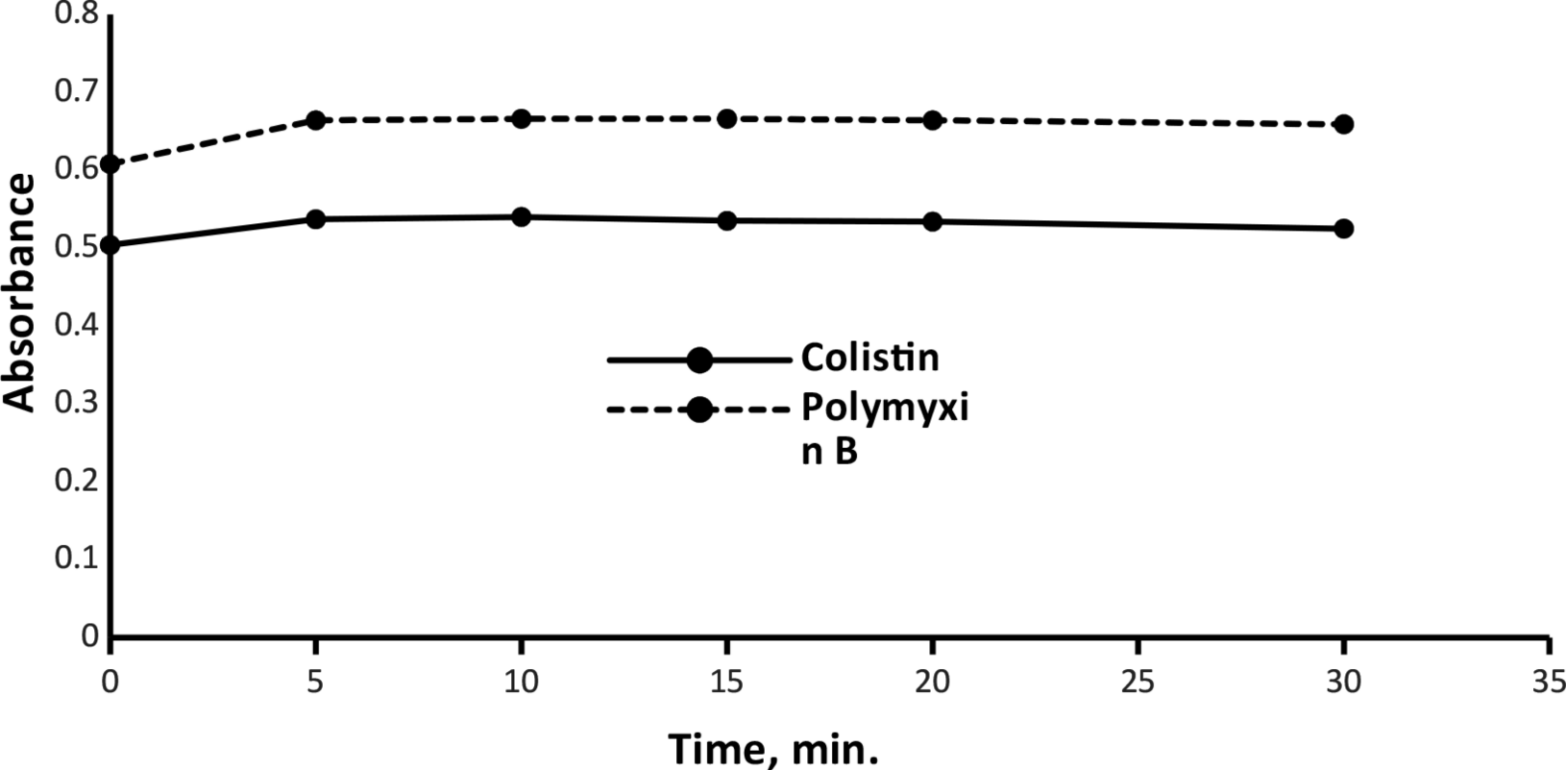
Effect of time on the absorbance of the ion-pair complexes of Colistin (3 µg mL^− 1^) and Polymyxin B (5 µg mL^− 1^)

Based on the screening of color intensity that stayed stable for more than one day, the influence of time and temperature on the yielded complex was investigated. Reaction time was tested up to 30 min, and a slight increase in ABS was noticed. Belongs to temperature, turbidity was induced at the elevated degree of temperature that resulted in a decrease in ABS values. Consequently, 10 min at room temperature was the optimum condition yielding the maximum ABS, as was observed in (Fig. 7).

#### Experimental solvent adjustment

**Fig. 8:**
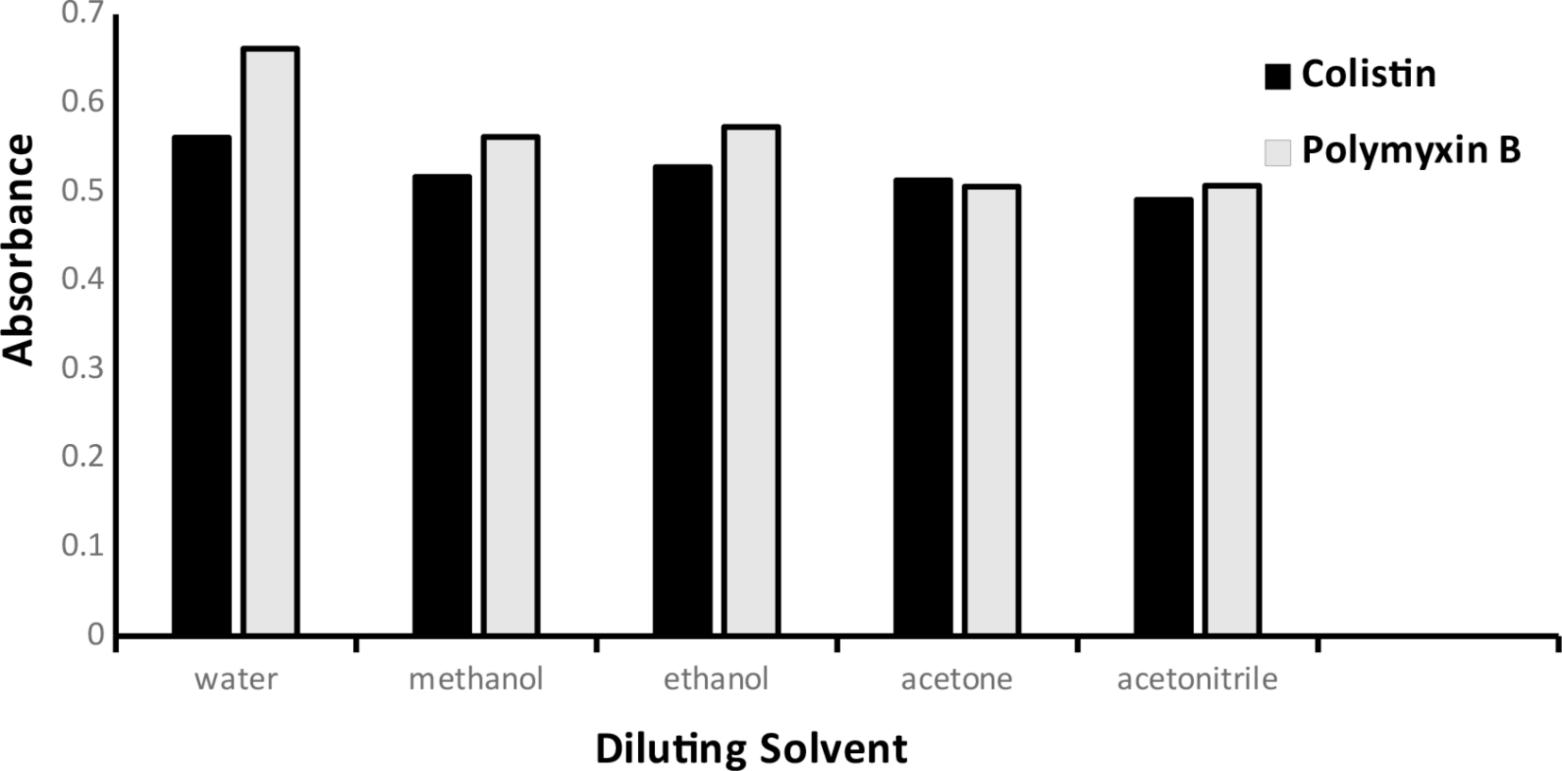
Effect of diluting solvent on the absorbance of the ion-pair complexes of Colistin (3 µg mL^− 1^) and Polymyxin B (5 µg mL^− 1^)

To demonstrate how different solvents affected the ABS values, numerous solvents including DW, acetonitrile, acetone, methanol, and ethanol were investigated. The best outcomes were obtained by employing DW as the diluting solvent that reflects the greenness of the presented work. All data were displayed in (Fig. 8).

#### Stoichiometric mole fraction method

**Fig. 9:**
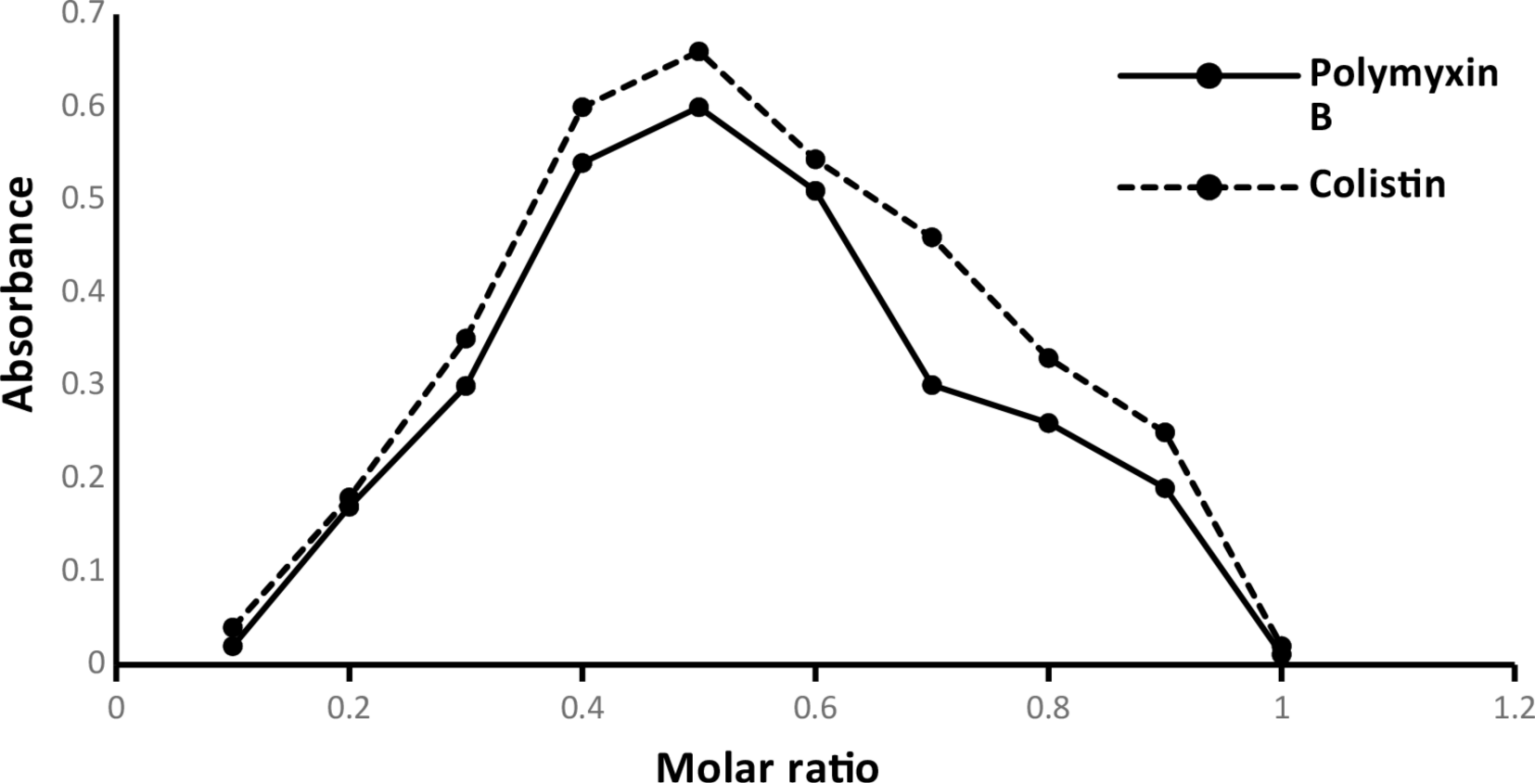
Job’s technique for determining the stoichiometry of the reaction using 3 × 10^− 4^ M concentration of both erythrosine B and Polymyxins

Equimolar (3 × 10^− 4^ M) standard solutions of CS, Poly B, and EB were used to implement Job’s method of continuous variations. The total volume of a series of complementary solutions was preserved at 1 mL. While the ratios of the examined drugs and EB were created as follows: (0.1:0.9, 0.2:0.8, 0.3:0.7, 0.4:0.6, 0.5:0.5, 0.6:0.4, 0.7:0.3, 0.8:0.2, and 0.9:0.1), then the general analytical methodology was implemented. As a result, the mole fraction of 0.5 outputted the highest results that revealed the molar ratio between the studied drugs and EB dye was 1:1. All details were illustrated in (Fig. 9).

### Validation study

In agreement with ICH guidelines [51], the validity of several analytical parameters was examined. Linearity and range, the limit of detection (LOD), the limit of quantification (LOQ), precision, accuracy, and robustness were examples of these screened factors.

#### Range of Linearity

By drawing the obtained absorbance against CS and Poly B at different concentrations (µg mL^− 1^), calibration curves were constructed. In the range of 1–6 and 1–9 µg mL^− 1^ for CS and Poly B, respectively, the relationship between the concentrations and the absorbance values was linear as indicated by high coorelation coefficient values (≥ 0.9990) and low standard deviation of residuals from line valus. All analytical parameters of both studied drugs that belong to the linear regression equation were gathered in (Table 1).

**Table 1:** Spectral features and validation parameters for the studied colorimetric approach

Parameters	Poly B	CS
λ_εξ_ (νµ)	545	
Linear range (µg mL^− 1^)	1–9	1–6
Correlation coefficient (r)	0.9995	0.9995
Determination coefficient (r^2^)	0.999	0.9991
Intercept ± SD*	0.3882 ± 3.671 × 10^− 3^	0.3745 ± 2.449 × 10^− 3^
Slope ± SD	0.0552 ± 6.52 × 10^− 4^	0.0534 ± 6.21 × 10^− 4^
Sy.x**	0.005053	0.00294
***LOD (µg mL^− 1^)	0.22	0.15
****LOQ (µg mL^− 1^)	0.66	0.46

The acceptability criteria for linearity is expressed as (r) and should be ≥ 0.9990

*SD: Standard Deviation ** Sy.x: Standard Deviation of residuals from lines

***LOD: Limit of detection. ****LOQ: Limit of quantitation

#### LOD & LOQ

LOD and \ LOQ were calculated to assess the sensitivity through the formulas 3.3 ϭ /S and 10 ϭ/S, respectively. Where ϭ is the standard deviation of the intercept and S is the calibration curve’s slope. The values for both cited drugs were gathered in (Table 1).

#### Accuracy

Three drug concentration levels (2, 4, and 6 µg mL^− 1^) were examined to evaluate the suggested method’s accuracy. The calculated percentage recovery (%R) showed a good agreement between the measured and actual values in its acceptable range (98–102%), proving the accuracy of the proposed method, as shown in (Table 2).

**Table 2:** Evaluation of the accuracy of the described spectrophotometric approach

	Poly B			CS	
Taken conc. (µg mL^− 1^)	Found conc. (µg mL^− 1^)	% Recovery* (%R)	Found conc. (µg mL^− 1^)	% Recovery (%R)	
2	1.987	99.35	2.098	98.60	
4	4.076	101.90	3.929	98.23	
6	5.923	98.72	6.055	100.92	
Mean*		99.99		99.25	
SD		1.68		1.46	
RSD		1.68		1.47	

% R: should be in the range (98–102%) * mean: of three replicate measurements

#### Precision

The closeness of the experimental values to each other was assessed in two ways. Firstly, intra-day precision, by replicating analysis of three different concentrations (2, 4, 6 µg mL^− 1^) of both experimented drugs at three different times along the day. Secondly, inter-day precision, by checking the same concentrations through successive three days. The calculated relative standard deviation (RSD) does not exceed 2, which is the accepted criteria, confirming the good precision of the described work. All data were illustrated in (Table 3).

**Table 3:** Intra- and inter-day precisions evaluation for PMS analysis

Concentration (µg mL^− 1^)	Poly B		CS	
	%Mean Recovery* ± RSD			
	Intra-day	Inter-day	Intra-day	Inter-day
2	99.76 ± 0.88	98.47 ± 1.12	101.61 ± 0.77	99.53 ± 0.72
4	100.13 ± 1.24	99.22 ± 0.69	99.51 ± 1.48	100.65 ± 1.74
6	98.44 ± 1.77	100.62 ± 0.38	98.16 ± 0.94	99.20 ± 0.42

RSD: relative standard deviation (should not exceed 2 ) * Mean of three determinations

#### Robustness

The method’s capacity to remain unaffected by small intentional changes to the experimental parameters without significant alterations to measure the absorbance values is referred to as robustness. Four factors from the experiment were screened: pH, buffer volume, dye volume, and reaction time. It was revealed that none of these factors has significantly affected. Thus, the established spectrophotometric approach was said to be robust and can be routinely employed in the assay of studied drugs. All outcomes were displayed in (Table 4).

**Table 4:** Robustness for determination of poly B and CS by the developed approach

Method Parameters	Poly B	CS
	%R* ± SD	
**pH**		
3.8	98.32 ± 1.87	99.93 ± 0.44
4	99.88 ± 0.91	101.54 ± 1.76
4.2	100.51 ± 1.65	98.74 ± 1.58
**Buffer volume**		
0.8	1.01.32 ± 0.99	99.05 ± 0.92
1	99.14 ± 0.87	100.04 ± 1.36
1.2	98.87 ± 1.36	98.11 ± 1.51
**Volume of erythrosine B**		
0.4	100.92 ± 0.80	98.80 ± 1.27
0.5	98.70 ± 1.35	99.09 ± 0.94
0.6	101.81 ± 1.79	101.71 ± 1.55
**Time of reaction (min)**		
5	101.44 ± 1.93	99.11 ± 1.59
10	99.71 ± 0.76	99.84 ± 0.52
15	97.99 ± 1.63	99.48 ± 0.99

% R: should be in the range (98–102%) * Mean of three determinations

### Applications of the developed approach

#### Assay of PMS in parenteral and oral dosage forms

Quantification of intravenous dosage forms of PMS and oral Colistin sulfate^®^ tablets was successfully implemented. Through statistically comparing the outcomes with the previously reported article [10], we used the Student’s t-test and the F-test to evaluate the precision and accuracy. Consequently, there was no major difference between the suggested approach and the published one. All values were inserted in (Table 5).

**Table 5:** Screening of PMS parenteral dosage forms by developed approach and compared with the reference approach

Market form	Developed method	Reference method		
	%*R* ± SD*		t-value**	f-value
Colomycin^®^ vial	100.05 ± 0.66	99.54 ± 1.57	0.851	2.233
Paximid^®^ vial	99.79 ± 1.04	99.62 ± 0.39	0.468	1.477

^*^Mean is the average of five determinations. ** Tabulated values at 95% confidence limit are t value = 2.306, F value = 6.338

#### Test of Content Homogeneity

The tested tablets exhibited the acceptable uniformity of CS, as shown by the estimated acceptance value (AV), which was 3.456 and was less than the highest permissible acceptable value of 15. All information was gathered in (Table 6).

**Table 6:** Content uniformity of CS in tablet form

Tablet number	Mean % R* of the claimed content Colistin sulfate ^®^ tablet
1	98.78
2	99.51
3	97.41
4	101.33
5	100.27
6	98.94
7	96.99
8	98.77
9	99.82
10	101.22
Mean	99.30
SD	1.44
RSD	1.45
Acceptance value (AV)	3.456
Maximum allowed AV (L1)	15

^*^Mean is the average of three determinations

## Greenness assessment

**Fig. 10:**
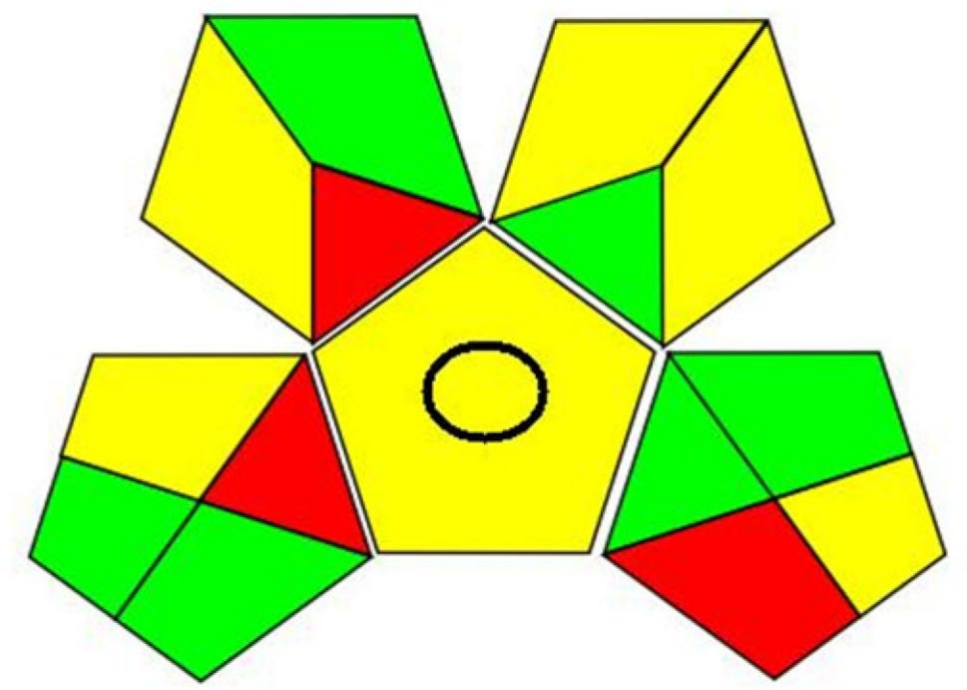
Pictogram of the developed method using GAPI tool

**Fig. 11:**
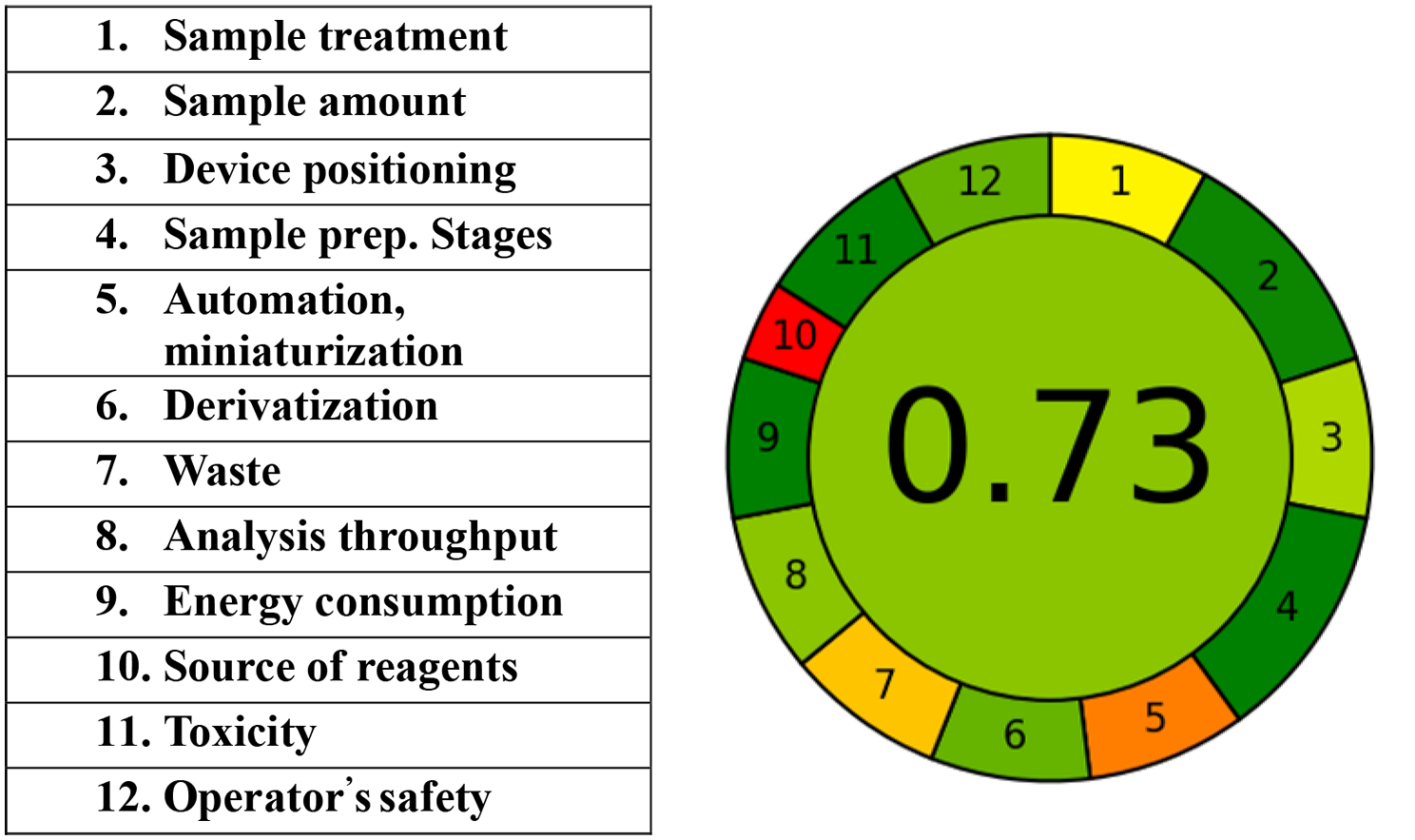
Pictogram of the developed method using AGREE tool

Many tools are now available to assess and contrast the greenness of different analytical techniques. GAPI (Green Analytical Procedure Index) tool has the unique advantage of covering the entire analytical method [52]. It consists of five pentagrams, each of which denotes a step in the analytical technique: sample preparation and collection; chemicals and solvents; applied equipment; and the goal of the analytical technique. Three color codes are used by GAPI: red denotes a significant environmental threat, while yellow and green denote a lower risk and increased greenness. AGREE (the Analytical GREEness) is also a flexible, and straightforward assessment tool that provides an easily interpretable and informative result [53]. The assessment criteria are taken from the 12 principles of green analytical chemistry and are transformed into a number. The result is a pictogram indicating the final score performance of the analytical procedure in each criterion and weights assigned by the user. All data were inserted in (Figs. 10 and 11). The good G score, 0.73, conflicted high greenness of the current work owing to utilizing distilled water throughout the procedures without extraction process and drastic conditions like heating [13, 14] or boiling [9, 11] or employing organic solvent [10], this is in light of comparison with the other reported fluorimetric techniques. On the other hand, not consuming large amounts of energy as in LC-MS articles or huge volumes of organic solvents like in HPLC published papers for the cited drugs, all previously mentioned factors negatively affected the G score. Recently, more than one method was employed for the assessment of the methodology such as cGAPI, AGREEprep, The up-to-date ChlorTox Scale indicator, CALIFICAMET tool, RGB12 algorithm, and spider diagram [54–57].

## Conclusion

The screened medications were quantified by employing a green, easy-to-implement, and economical spectrophotometric approach. The produced complexes were water-soluble, therefore there was no need for organic solvents, making the methodology safer for the environment. Furthermore, the suggested approach is more efficient in terms of time reliability, and sensitivity than other reported spectrophotometric methods. In light of this, the described approach can be easily implemented for the routine assay of the studied drugs instead of the previously published spectroscopic and chromatographic techniques in quality control laboratories. Moreover, Content uniformity testing for CS tablets was employed and the described current work was considered the first colorimetric method employed to quantify the intravenous market forms of the cited drug.

## Data Availability

No datasets were generated or analysed during the current study.
